# CD80 down-regulation is associated to aberrant DNA methylation in non-inflammatory colon carcinogenesis

**DOI:** 10.1186/s12885-016-2405-z

**Published:** 2016-07-04

**Authors:** Marco Scarpa, Melania Scarpa, Ignazio Castagliuolo, Francesca Erroi, Silvia Basato, Paola Brun, Imerio Angriman, Carlo Castoro

**Affiliations:** Esophageal and Digestive Tract Surgery Unit, Veneto Institute of Oncology IOV - IRCCS, Padova, Italy; Department of Molecular Medicine, University of Padova, Padova, Italy; Department of Surgery, Oncology and Gastroenterology DISCOG, University of Padova, Padova, Italy

## Abstract

**Background:**

The lack of positive costimulatory molecules represents one of the mechanisms by which tumor cells evade immune surveillance. Promoter hypermethylation plays a major role in cancer development through transcriptional silencing of critical genes. The aim of this study was to examine the expression of the costimulatory molecule CD80 in relationship with genomic methylation in non-inflammatory colon carcinogenesis.

**Methods:**

Colonic mucosal samples were collected from healthy subjects (*n* = 30) and from dysplastic adenoma (*n* = 14), and colon adenocarcinoma (*n* = 10). DNA methyltransferases-1, −3a, −3b and CD80 mRNA expression were quantified by real time qRT-PCR. The methylation status of CDH13, APC, MLH1, MGMT1 and RUNX3 gene promoters was assessed by methylation-specific PCR. CD80 expression was assessed in HT29, HCT-15 and LoVo cell lines after treatment with the DNA-methyltransferase inhibitor 5-Aza-2′-deoxycytidine.

**Results:**

CD80 mRNA levels were significantly lower in the non-inflammatory dysplastic colonic mucosa of patients with one or more methylated genes and inversely correlated with patients’ methylation scores (τ = −0.41, *p* = 0.05 and τ = −0.37, *p* = 0.05, respectively). Treatment with 5-Aza-2′-deoxycytidine significantly increased CD80 expression both in terms of the level of CD80 mRNA (*p* = 0.007) and of CD80+ cells (*p* = 0.003).

**Conclusions:**

These results indicate that the failure of immune surveillance mechanisms in non-inflammatory colon carcinogenesis may be linked to genomic methylation directly or indirectly affecting CD80 expression.

**Electronic supplementary material:**

The online version of this article (doi:10.1186/s12885-016-2405-z) contains supplementary material, which is available to authorized users.

## Background

Immune surveillance of nascent cancer cells is a fundamental process mediated by T-cells, macrophages, and natural killer cells that work to protect host against the unrestrained proliferation of transformed cells [1]. Full T-cell activation requires the engagement of T-cell receptors with specific major histocompatibility complexes at the surface of antigen-presenting cells in the presence of adequate co-stimulatory signals provided by CD80 or CD86 [2]. Oncogenic insults inducing CD80 expression seem then to be able to modulate the anti-tumor immune response [3]. The significant overexpression of CD80 in the colonic mucosa of patients with ulcerative colitis (UC) and dysplasia as opposed to CD80 down-regulation in non-inflammatory colon cancer [4–6] have led to the hypothesis that the lack of positive co-stimulatory molecules is one of the main mechanisms by which colorectal cancer (CRC) escapes immune surveillance [7]. The molecular mechanisms underlying immune surveillance remain, nevertheless, largely unknown.

An altered methylation pattern in cancer cell genomes is a well recognized characteristic of tumor cells, and specific aberrant methylation events take place during the early stages of colorectal carcinogenesis leading to profound modifications in gene expression [8]. The aberrant methylation of H-cadherin (CDH13) commencing at an early stage of colorectal tumorigenesis frequently silences, in fact, the expression of this tumor suppressor gene in colorectal adenomas and cancers [9]. Besides germ-line mutations associated with hereditary familial adenomatous polyposis and somatic mutations in sporadic colorectal tumors, hypermethylation also provides an important mechanism underlying impaired APC function [10]. Moreover, the hypermethylation of the CpG island within the DNA-repair protein O-6-methylguanine-DNA-methyltransferase (MGMT) gene [11] and in the MLH1 gene is associated with a reduced gene expression observed in the majority of sporadic primary CRC with microsatellite instability [12]. Finally, RUNX3 hypermethylation decreases TGF-β/BMP signaling in gastrointestinal cancer cells [13].

In addition to oncogenes or oncosuppressors, some immune stimulatory molecules are regulated in cancer cells by DNA methylation in their promoter regions. MHC class I and its antigen presentation machinery have been shown to be regulated by DNA methylation [14–17]. Adhesion molecules [14–18], such as ICAM-1 and LFA-3, and costimulatory molecules [17, 18], such as CD40 and CD86, can be regulated by DNA methylation in cancer cells. Thus, demethylation treatment can dramatically increase the susceptibility of some tumor cells to T-cell destruction [15, 19–21]. Our first step in investigating the role of DNA methylation in CRC immune surveillance consisted in examining the expression of CD80; we then went on to analyze its relationship with genomic methylation during the early stages of colon carcinogenesis.

## Methods

### Patients

A prospective cohort study of healthy controls (*n* = 30) and patients (*n* = 24) who underwent colonoscopy for screening or post-operative follow-up or colonic resection for colorectal cancer was designed. Biopsy samples of healthy mucosa (*n* = 30), of normal and diseased mucosa from patients with dysplastic adenoma (*n* = 14), and from patients with colon adenocarcinoma (*n* = 10, one patients staged T1N0M0, two T2N0M0, two T3N0M0, four T3N1M0 and one T3N1M1) were collected. Patients’ and controls’ characteristics are outlined in Table 1.

**Table 1:** Patients characteristics

Non-inflammatory carcinogenesis	Healthy controls	Adenoma and dysplasia	Cancer
Patients (*n*)	30	14	10
median age (range)	59.5 (52–69) years	61 (51–69) years	63 (49–74) years
gender (male/female)	14:16	7:7	7:3
procedures	colonoscopy: 30	colonoscopy: 12	colonic resection: 8
		RPC: 2	rectal resection: 2
carcinogenesis stage	NA	LGD: 9	T1N0M0: 1
		HGD: 5	T2N0M0: 2
			T3N0M0: 2
			T3N1M0: 4
			T3N1M1: 1

*HGD* high grade dysplasia, *LGD* low grade dysplasia, *RPC* restorative proctocolectomy

### Gene expression analysis

Total RNA was extracted using the RNeasy Plus Kit (Qiagen) according to the manufacturer’s protocol. At that point 0.5 μg total RNA was converted to cDNA using the Applied Biosystems cDNA Synthesis kit, again, according to the manufacturer’s instructions. DNA methyltransferase-1, −3a, −3b (DNMT-) and CD80 mRNA expression was quantified by real time qRT-PCR. Specific mRNA transcripts were quantified with SYBR Green PCR Master Mix in a ABI PRISM 7000 Sequence Detection System (Applied Biosystems). ACTB expression was used as reference gene for normalization. Primer sequences and PCR conditions are outlined in Table 2.

**Table 2:** Real-Time qPCR and Methylation-Specific PCR primers

Real-Time qPCR primers				
Gene	NCBI ref seq	Sequence 5'-- > 3'	Ta, °C	Amplicon, bp
Actb	NM_001101	fw CTGGACTTCGAGCAAGAGATG	60	180
		rv AGTTGAAGGTAGTTTCGTGGATG		
Cd80	NM_005191	fw CTCACTTCTGTTCAGGTGTTATCCA	62	121
		rv TCCTTTTGCCAGTAGATGCGA		
Dnmt1	NM_001130823	fw TACCTGGACGACCCTGACCTC	60	103
		rv CGTTGGCATCAAAGATGGACA		
Dnmt3a	NM_175629	fw GACAAGAATGCCACCAAAGC	60	190
		rv CGTCTCCGAACCACATGAC		
Dnmt3b	NM_006892	fw GGCAAGTTCTCCGAGGTCTCTG	60	113
		rv TGGTACATGGCTTTTCGATAGGA		
Methylation specific PCR primers				
Gene	sequence 5'-- > 3'		Ta,°C	Amplicon, bp
CDH13 meth	Fw TCGCGGGGTTCGTTTTTCGC		66	243
	Rv GACGTTTTCATTCATACACGCG			
CDH13 unmeth	Fw TTGTGGGGTTGTTTTTTGT		55	242
	Rv AACTTTTCATTCATACACACA			
APC meth	Fw TATTGCGGAGTGCGGGTC		64	98
	Rv TCGACGAACTCCCGACGA			
APC unmeth	Fw GTGTTTTATTGTGGAGTGTGGGTT		62	108
	Rv CCAATCAACAAACTCCCAACAA			
RUNX3 meth	Fw TTACGAGGGGCGGTCGTACGCGGG		71	220
	Rv AAAACGACCGACGCGAACGCCTCC			
RUNX3 unmeth	Fw TTATGAGGGGTGGTTGTATGTGGG		64	220
	Rv AAAACAACCAACACAAACACCTCC			
MGMT meth	Fw TTTCGACGTTCGTAGGTTTTCGC		64	81
	Rv GCACTCTTCCGAAAACGAAACG			
MGMT unmeth	Fw TTTGTGTTTTGATGTTTGTAGGTTTTTGT		64	93
	Rv AACTCCACACTCTTCCAAAAACAAAACA			
MLH1 meth	Fw ACGTAGACGTTTTATTAGGGTCGC		56	115
	Rv CCTCATCGTAACTACCCGCG			
MLH1 unmeth	Fw TTTTGATGTAGATGTTTTATTAGGGTTGT		56	124
	Rv ACCACCTCATCATAACTACCCACA			

### Methylation specific PCR

Genomic DNA was extracted from tissues using a DNeasy Blood & Tissue Kit (Qiagen) according to the manufacturer’s directions. Sodium bisulfate modification of gDNA was performed using the EZ DNA Methylation-Gold Kit (Zymo Research) following the manufacturer’s instructions. The primers for APC, CDH13, MGMT, MLH1 and RUNX3 methylation-specific PCR and PCR conditions are outlined in Table 2. The EpiTect PCR Control DNA Set (Qiagen) was used as the positive control for the methylated and unmethylated genes. Each PCR was done in a final volume of 25 μL containing 10 ng of bisulfite converted gDNA, 1× PCR buffer, 0.25 mmol/L deoxynucleotide triphosphate, 400 nmol/L each primer, and 1 unit ZymoTaq (Zymo Research). PCR amplification was done as follows: 95 °C for 10 min followed by 40 cycles at 95 °C for 30 s, the specific annealing temperature for each gene for 30 s and 72 °C for 30s; and, in a final extension step, at 72 °C for 7 min. PCR products were resolved by 3 % agarose gel electrophoresis and each case was scored as methylated or unmethylated. Since we aimed to investigate the role of any grade of methylation, we considered as methylated those sample showing any detectable specific band. The patients’ global methylation scores were calculated by summing the number of methylated genes (range 0–5).

### CD80 expression in intestinal epithelial cell lines

Three colorectal adenocarcinoma cell lines were used in the present study. HCT-15, HT-29 and LoVo were purchased from the American Tissue Culture Collection, cultured in medium (DMEM for HCT-15 and HT29, Ham’s F12 Nutrient Mixture for LoVo) supplemented with 10 % FBS and 1X pen/strep solution (all from Life Technologies) and maintained in humidified 37 °C 5 % CO_2_ incubators according to the manufacturer’s protocol. Fifty percent confluent cells were incubated with the DNA methyltransferase inhibitor 5-Aza-2′-deoxycytidine (5AZAdC) (5 μM, Sigma) or vehicle as a control for 96 h. Cells were harvested for Real-time qPCR or flow cytometric analysis.

### Flow cytometry

HT29, HCT-15 and LoVo cells were stained with CD80-FITC antibody (clone 2D10, eBioscience) in staining buffer (PBS with 1 % FBS) on ice for 30 min. Flow cytometric analysis was performed by a FACScalibur flow cytometer using CellQuest software (Becton Dickinson).

### Statistical analysis

Data are shown as mean ± SEM. Non-parametric Mann–Whitney’s U-test was carried out to compare independent variables, the Wilcoxon test was used to compare matched variables, and the Kruskal-Wallis ANOVA was performed to compare multiple variables. The Kendall rank correlation test was applied. Differences were considered significant at *p* < 0.05.

## Results

### Methylation status of gene promoters involved in the early stages of colon carcinogenesis

**Fig. 1:**
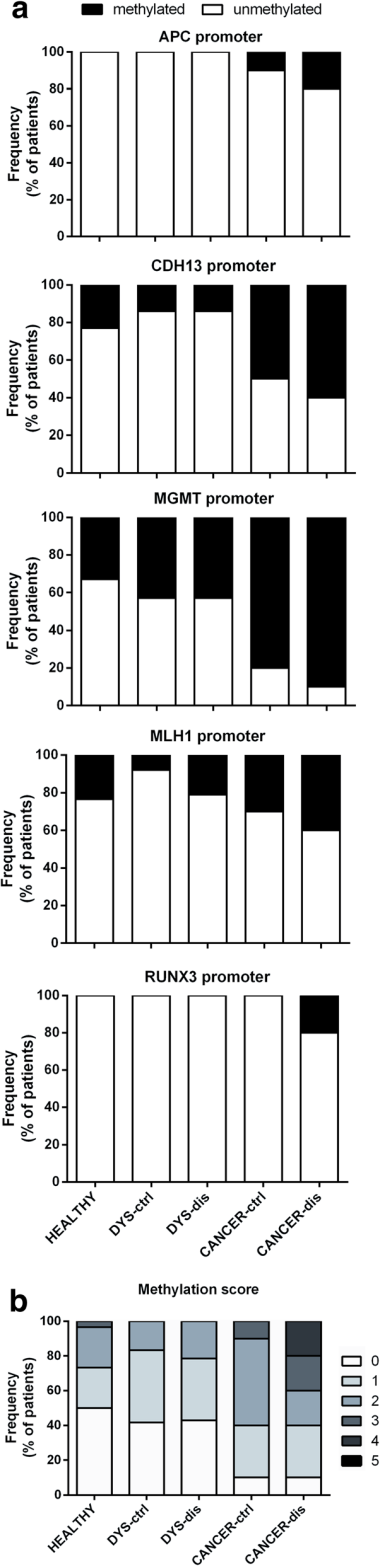
Methylation status of gene promoters involved in the early stages of colon carcinogenesis. **a** The methylation status of APC, CDH13, MGMT, MLH1 and RUNX3 gene promoters was assessed by methylation specific-PCR of colonic mucosa specimens derived from patients with colon carcinogenesis. The frequency of methylation of each gene among the different patients groups is shown. **b** The frequency of total methylation, defined as the sum of the methylated genes detected (range 0–4), among the different patients groups is shown. *CTRL*, healthy control; *DYS*, dysplasia and adenoma

The promoter methylation status of five genes known to be associated with early stages of colon carcinogenesis was assessed in the colonic mucosa of 54 subjects (Table 1). They consisted of 30 healthy controls, 14 with adenoma and dysplasia, and 10 with colorectal adenocarcinoma. The promoter regions of APC, CDH13, MGMT1 and RUNX3 were more frequently methylated in patients with invasive adenocarcinoma (*p* = 0.04, *p* = 0.02, *p* = 0.03 and *p* = 0.05, respectively) (Fig. 1a). Consequently, the methylation score was significantly higher in patients with cancer than those with pre-neoplastic lesions (i.e. adenomas) and healthy subjects (*p* = 0.04) (Fig. 1b).

### CD80 and DNMTs mRNA expression along colonic carcinogenesis pathway

**Fig. 2:**
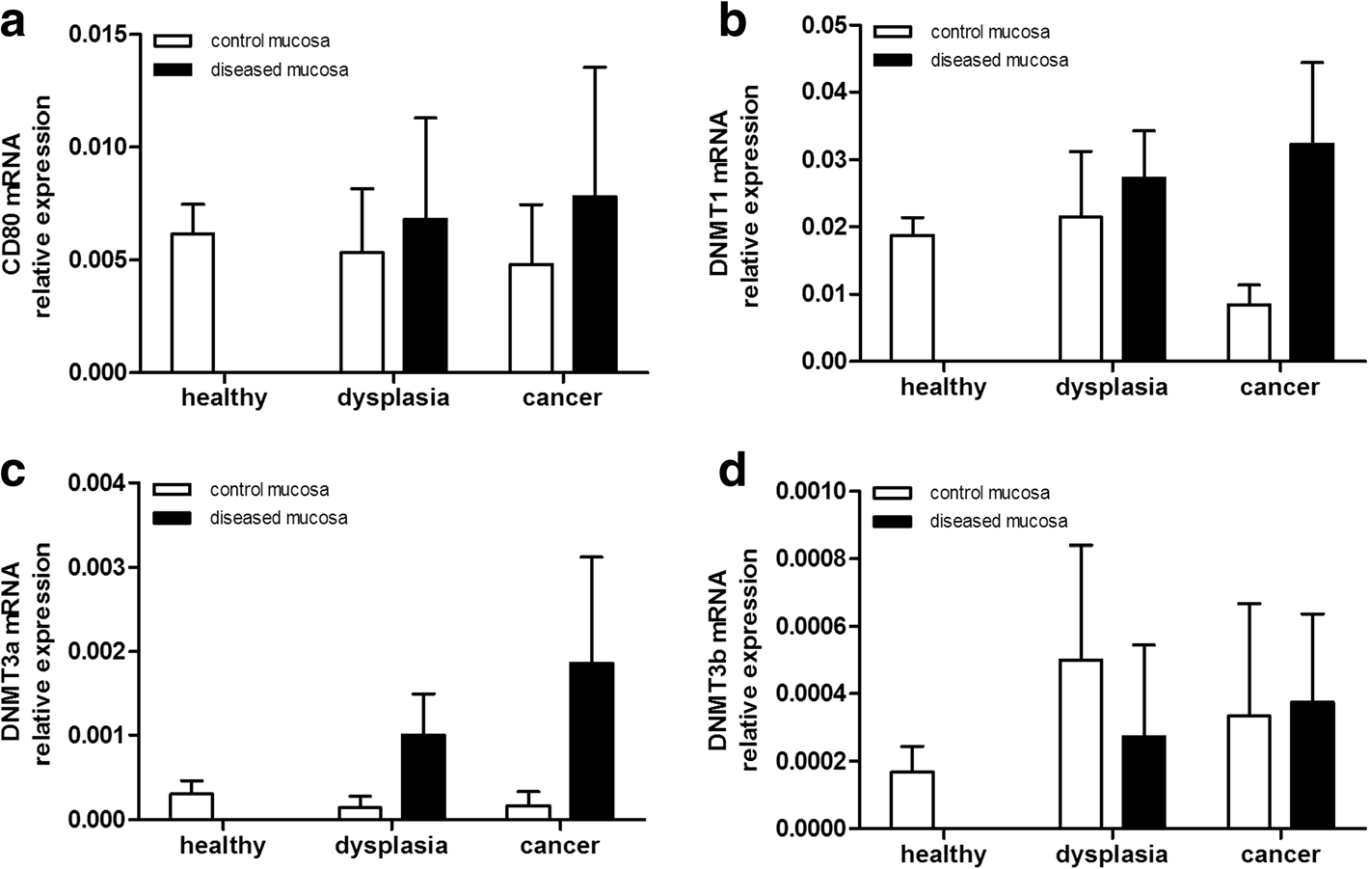
CD80 and DNA methyltransferases mRNA levels in colon carcinogenesis. **a** CD80, (**b**) DNMT1, (**c**) DNMT3a and (**d**) DNMT3b mRNA levels were quantified by Real Time qPCR in the colonic mucosa specimens of patients with non-inflammatory colon carcinogenesis; mRNA mean levels ± SEM in the different patients groups are shown. Kruskal-Wallis ANOVA test was performed to compare multiple groups. *CTRL*, control; *DYS*, dysplasia and adenoma

The expression of CD80 and methyltransferase (DNMT)-1, −3a, and −3b was quantified in the different steps of sporadic carcinogenesis. CD80 mRNA expression did not show any significant variation as shown in Fig. 2a. On the other hand, in colonic lesions the expression of all three DNMTs tended to increase progressively along the carcinogenesis pathways, as shown in Fig. 2b-d.

### Relationship between the methylation status, DNMTs and CD80 expression in colon carcinogenesis

**Fig. 3:**
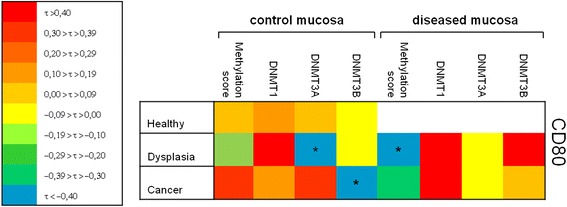
Relationship between the methylation status and CD80 in colon carcinogenesis. Correlation analysis of CD80 mRNA expression with DNMTs expression and DNA methylation status. The heat-map depicts correlation coefficient τ calculated using Kendall rank analysis. *p < 0.05

As shown in Fig. 3, CD80 mRNA levels were inversely correlated with the DNMT3A mRNA levels in healthy colonic mucosa of patients with adenoma and with DNMT3B in healthy colonic mucosa of patients with cancer (τ = −0.44, *p* = 0.07 and τ = −0.50, *p* = 0.04, respectively). Moreover, in colonic adenoma CD80 mRNA levels resulted inversely correlated to the number of methylated genes (τ = −0.43, *p* = 0.04).

### DNA methylation affects CD80 expression in intestinal epithelial cells

**Fig. 4:**
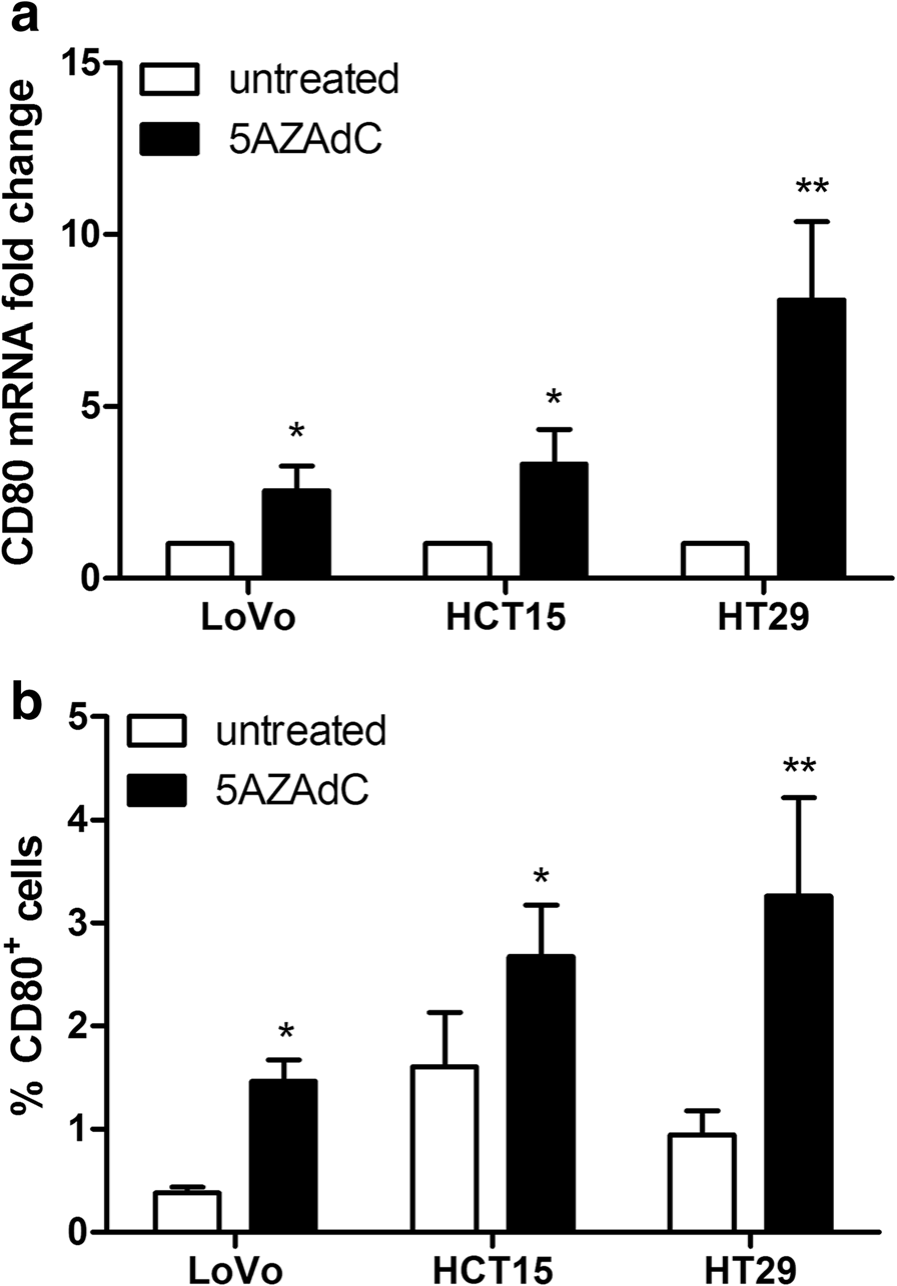
DNMTs inhibition triggers CD80 expression in intestinal epithelial cells. LoVo, HCT15 and HT29 cells were cultered in the presence or not of the DNMTs inhibitor 5AZAdC for 96 h, then cells were harvested for analysis. **a** Cd80 mRNA was quantified by Real Time qPCR and (**b**) CD80 protein expression was evaluated by flow cytometry. The mean ± SEM is represented (*n* = 8)

To assess the impact of DNA methylation on CD80 gene expression levels in intestinal cancer cells, HT29, HTC15 and LoVo cell lines were treated with 5AZAdC, the DNMTs inhibitor. As shown in Fig. 4a and b, at basal levels CD80 mRNA was barely detectable and the percentage of CD80^+^ HT29, HTC15 and LoVo cells was < 2 %. Adding 5AZAdC to HT29, HTC15 and LoVo cells coltures significantly increased CD80 expression both in terms of CD80 mRNA levels (*p* = 0.005, *p* = 0.02 and *p* = 0.02, respectively) and of CD80^+^ cells (*p* = 0.001, *p* = 0.02 and *p* = 0.04, respectively).

## Discussion

Cancer immune surveillance is a fundamental process by which immune cells protect against cancer formation by identifying and eliminating tumor cells on the basis of their expression of tumor-specific and stress-induced antigens [1]. While it has been suggested that the lack of positive co-stimulatory molecules is the mechanism by which tumor cells evade immune surveillance, the molecular events underlying this process remain elusive.

In this study, we show that CD80 mRNA expression, the co-stimulatory molecule found in colon carcinogenesis, inversely correlates with the inducible DNMTs expression in healthy tissue of patients with adenoma or cancer and with the number of genes that have promoters which are often aberrantly hypermethylated at early stages of colorectal tumorigenesis and, as a consequence, thought to contribute to CRC pathogenesis [8]. Indeed, APC, CDH13, MGMT1 and RUNX3 methylation frequency of gene promoters as well as DNMTs expression progressively increases along the pathway towards invasive cancer in sporadic colonic carcinogenesis. Taken together these data suggest that DNA methylation plays a role in CD80 expression. This association has been found to be notable at the dysplastic stage of carcinogenesis when the CD80-driven immune surveillance process might be critical in preventing cancer cells from escaping [6, 22]. But although two prospective studies characterized by adequate follow-up and precise definition of the adenoma site failed to show complete regression or spontaneous reduction of polyps [23, 24], the findings of a more recent epidemiological investigation suggest that dysplastic adenoma prevalence in humans is caused by a dynamic process including both adenoma formation and regression [25]. Our data are, indeed, the first to suggest that DNA methylation of CD80 gene may explain why immune surveillance mechanisms can at times fail in patients with non-inflammatory colorectal carcinogenesis.

Interestingly, a recent investigation reported hypermethylation of the CD80 promoter in mice tumours, and treatment with decitabine, a DNA methyltransferase inhibitor, was found to enhance CD80 expression in EL4 cells via demethylation of CpG dinucleotide sites in the promoter of CD80 gene resulting in T lymphocyte infiltration into tumors and, ultimately, in tumor rejection [26]. Likewise, epigenetic silencing of CD70, a CD80 related co-stimulatory molecule, by DNA methylation was noted during breast cancer progression in an *in vitro* model [27].

Our *in vitro* assay on HT29, HTC15 and LoVo CRC cell lines further supports the hypothesis that DNA methylation is involved in the inhibition of the gene expression of CD80. There was, in fact, a 10-fold increase in CD80 mRNA levels and the number of CD80+ cells was doubled following treatment with a DNA methyltransferase inhibitor. These data are particularly relevant because they confirm the hypothesis of a role of gene methylation in the inhibition of CD80 expression in three different CRC cell lines.

A limit of the present study is the lack of a direct evidence of DNA methylation of the CD80 promoter. Usually, but not exclusively, methylation occurs at the so-called CpG islands and if hypermethylation involves the promoter region it may result in gene silencing. CD80 promoter do not contain any sequence with characteristics making methylation easy to detect (CD80 at chr3:119524293-119559634 Genome Browser http://genome.ucsc.edu/index.html). Given the difficulty in directly assessing whether and at what stage CD80 methylation takes place along the colorectal carcinogenesis pathway and in order to assess if hypermethylation plays a role in CD80 down-regulation in human colorectal tumorigenesis, we examined the correlation between expression of CD80 and the methylation of genes involved in the early stages of CRC carcinogenesis and the expression of DNMTs and attempted to verify our hypothesis by developing an *in vitro* assay. Finally, this study did not explore the interactions of CD80 and PD-1 with B7H1 (programmed death ligand 1 [PD-L1]) [28]. In fact, although they are both located on antigen presenting cells’ surface, CD80 also has an appreciable affinity for the PD-L1 [29]. Their interplay has a pivotal role in controlling T cell activation, proliferation, anergy, and apoptosis and methylation might play a role in their regulation. However, the interactions between the two pathways remain still unknown.

## Conclusion

In conclusion, gene methylation seems to be associated to the CD80 down-regulation observed in dysplasia of sporadic colonic carcinogenesis. In vitro inhibition of DNA methyltransferases significantly enhances CD80 expression in colon cancer cells. We hypothesize that the failure of immune surveillance mechanisms in colonic non-inflammatory carcinogenesis is linked to genomic methylation directly or indirectly affecting CD80 expression.

## Abbreviations

5AzadC, 5-Aza-2′-deoxycytidine; APC, Adenomatous Polyposis Coli; CDH13, H-cadherin; CRC, colorectal cancer; DNMT, DNA methyltransferase; MGMT, O-6-methylguanine-DNA methyltransferase; MLH1, MutL homolog 1; RUNX3, Runt-related transcription factor 3; UC, ulcerative colitis

## Additional files

Additional file 1: Figure S1. MSP analysis. Representative results of methylation-specific PCR of APC, CDH13, MGMT, MLH1 and RUNX3 in colorectal mucosa specimen. The presence of a visible PCR product indicates the presence of unmethylated and methylated genes as shown; the EpiTect PCR Control DNA Set (Qiagen) was used as the positive control (+) for the methylated and unmethylated genes. (ZIP 1770 kb) Additional file 2: Dataset for CD80 and methylation privacyR3. (XLS 48 kb)
