# Supplementary figures and images for: CD80 down-regulation is associated to aberrant DNA methylation in non-inflammatory colon carcinogenesis
Source: BMC Cancer. 2016 Jul 4;16:388. doi: 10.1186/s12885-016-2405-z (PMC4932699; doi:10.1186/s12885-016-2405-z)

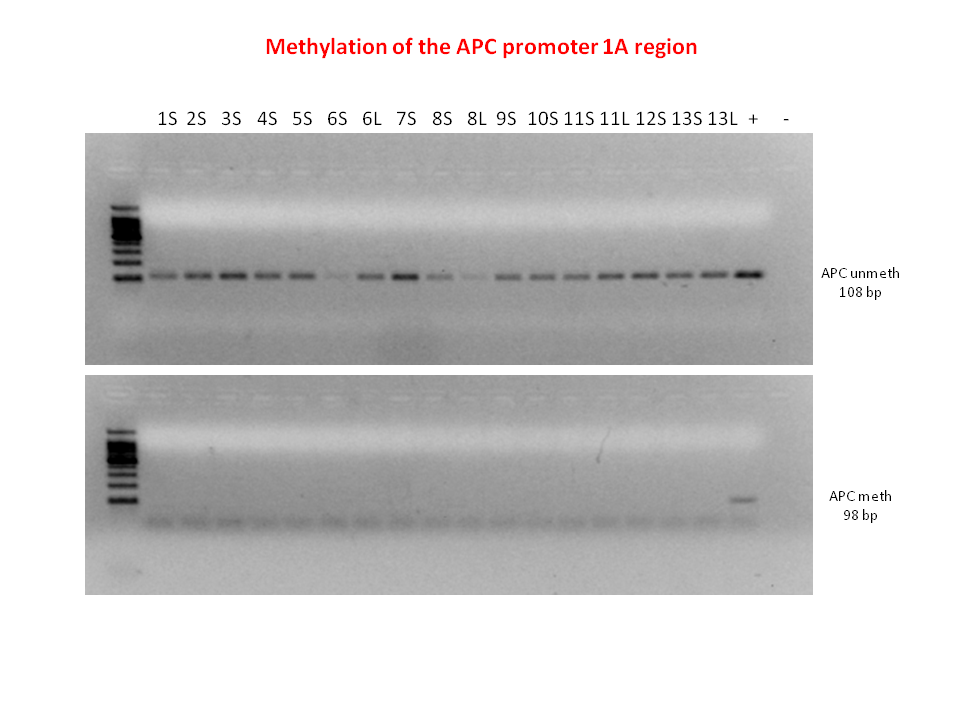

Supplement: Additional file 1: Figure S1. — MSP analysis. Representative results of methylation-specific PCR of APC, CDH13, MGMT, MLH1 and RUNX3 in colorectal mucosa specimen. The presence of a visible PCR product indicates the presence of unmethylated and methylated genes as shown; the EpiTect PCR Control DNA Set (Qiagen) was used as the positive control (+) for the methylated and unmethylated genes. (ZIP 1770 kb) [file 12885_2016_2405_MOESM1_ESM.zip › suppl. fig. 1aR3.TIF]

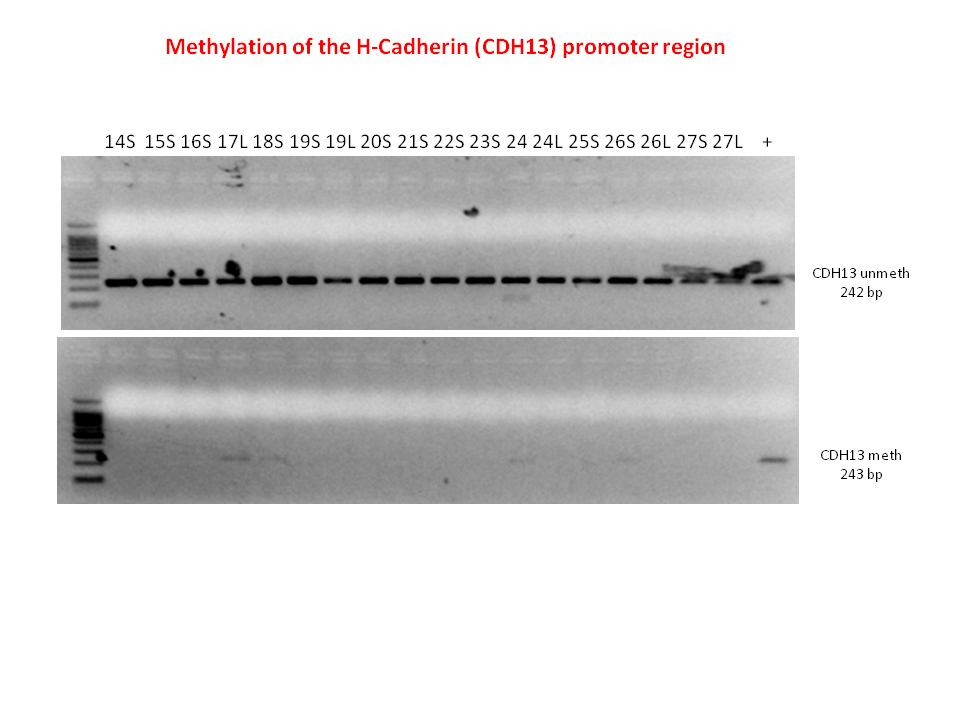

Supplement: Additional file 1: Figure S1. — MSP analysis. Representative results of methylation-specific PCR of APC, CDH13, MGMT, MLH1 and RUNX3 in colorectal mucosa specimen. The presence of a visible PCR product indicates the presence of unmethylated and methylated genes as shown; the EpiTect PCR Control DNA Set (Qiagen) was used as the positive control (+) for the methylated and unmethylated genes. (ZIP 1770 kb) [file 12885_2016_2405_MOESM1_ESM.zip › suppl. fig. 1bR3.TIF]

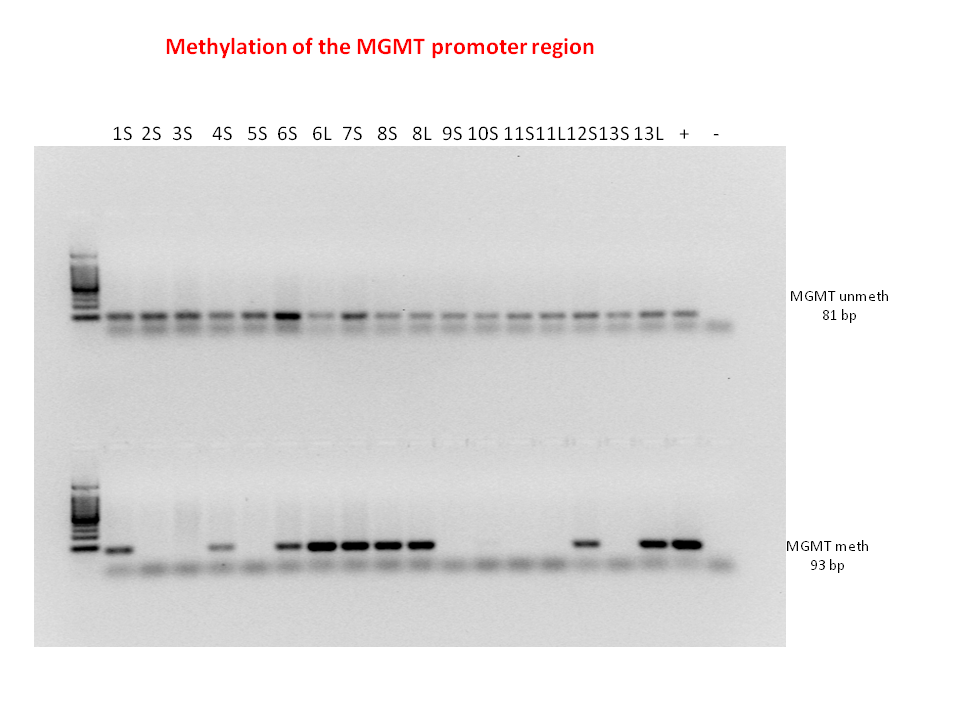

Supplement: Additional file 1: Figure S1. — MSP analysis. Representative results of methylation-specific PCR of APC, CDH13, MGMT, MLH1 and RUNX3 in colorectal mucosa specimen. The presence of a visible PCR product indicates the presence of unmethylated and methylated genes as shown; the EpiTect PCR Control DNA Set (Qiagen) was used as the positive control (+) for the methylated and unmethylated genes. (ZIP 1770 kb) [file 12885_2016_2405_MOESM1_ESM.zip › suppl. fig. 1cR3.TIF]

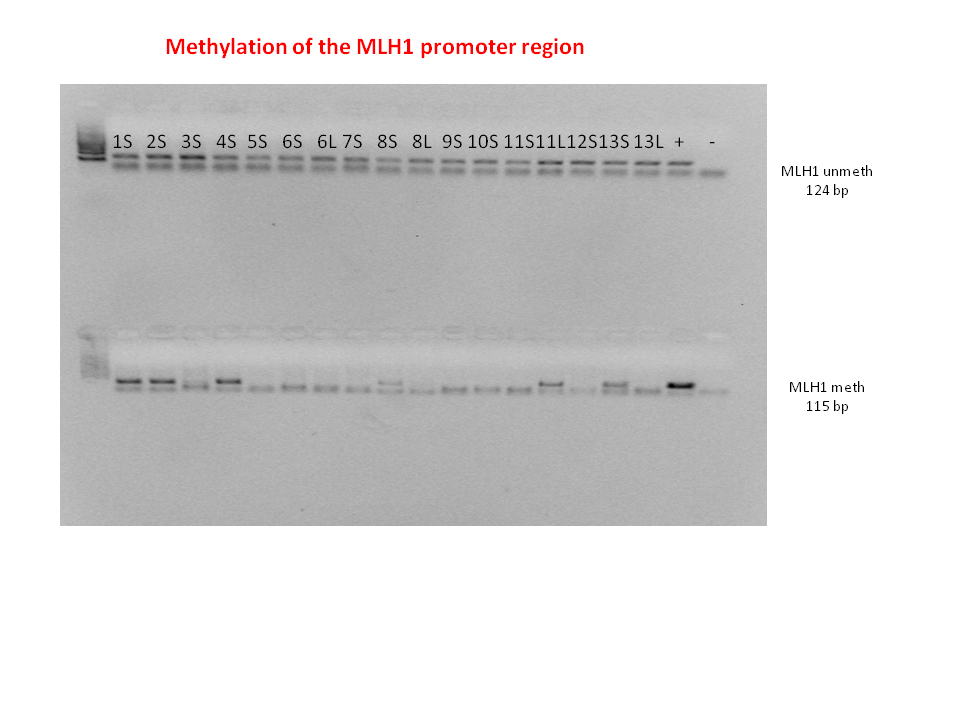

Supplement: Additional file 1: Figure S1. — MSP analysis. Representative results of methylation-specific PCR of APC, CDH13, MGMT, MLH1 and RUNX3 in colorectal mucosa specimen. The presence of a visible PCR product indicates the presence of unmethylated and methylated genes as shown; the EpiTect PCR Control DNA Set (Qiagen) was used as the positive control (+) for the methylated and unmethylated genes. (ZIP 1770 kb) [file 12885_2016_2405_MOESM1_ESM.zip › suppl. fig. 1dR3.TIF]

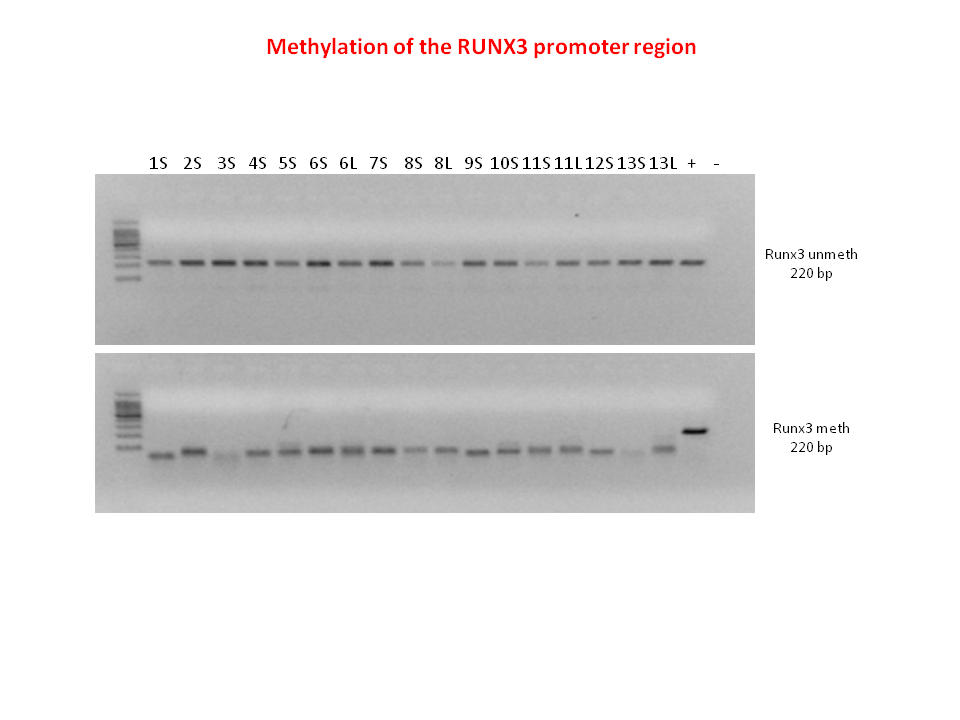

Supplement: Additional file 1: Figure S1. — MSP analysis. Representative results of methylation-specific PCR of APC, CDH13, MGMT, MLH1 and RUNX3 in colorectal mucosa specimen. The presence of a visible PCR product indicates the presence of unmethylated and methylated genes as shown; the EpiTect PCR Control DNA Set (Qiagen) was used as the positive control (+) for the methylated and unmethylated genes. (ZIP 1770 kb) [file 12885_2016_2405_MOESM1_ESM.zip › suppl. fig. 1eR3.TIF]
